# Biased Signaling of Protease-Activated Receptors

**DOI:** 10.3389/fendo.2014.00067

**Published:** 2014-05-13

**Authors:** Peishen Zhao, Matthew Metcalf, Nigel W. Bunnett

**Affiliations:** ^1^Monash Institute of Pharmaceutical Sciences, Parkville, VIC, Australia; ^2^Department of Pharmacology, University of Melbourne, Melbourne, VIC, Australia

**Keywords:** PARs, proteases, biased signaling, G proteins, β-arrestins, signal transduction

## Abstract

In addition to their role in protein degradation and digestion, proteases can also function as hormone-like signaling molecules that regulate vital patho-physiological processes, including inflammation, hemostasis, pain, and repair mechanisms. Certain proteases can signal to cells by cleaving protease-activated receptors (PARs), a family of four G protein-coupled receptors. PARs are expressed by almost all cell types, control important physiological and disease-relevant processes, and are an emerging therapeutic target for major diseases. Most information about PAR activation and function derives from studies of a few proteases, for example thrombin in the case of PAR_1_, PAR_3_, and PAR_4_, and trypsin in the case of PAR_2_ and PAR_4_. These proteases cleave PARs at established sites with the extracellular N-terminal domains, and expose tethered ligands that stabilize conformations of the cleaved receptors that activate the canonical pathways of G protein- and/or β-arrestin-dependent signaling. However, a growing number of proteases have been identified that cleave PARs at divergent sites to activate distinct patterns of receptor signaling and trafficking. The capacity of these proteases to trigger distinct signaling pathways is referred to as biased signaling, and can lead to unique patho-physiological outcomes. Given that a different repertoire of proteases are activated in various patho-physiological conditions that may activate PARs by different mechanisms, signaling bias may account for the divergent actions of proteases and PARs. Moreover, therapies that target disease-relevant biased signaling pathways may be more effective and selective approaches for the treatment of protease- and PAR-driven diseases. Thus, rather than mediating the actions of a few proteases, PARs may integrate the biological actions of a wide spectrum of proteases in different patho-physiological conditions.

## Introduction

With over 800 members in mammals, G protein-coupled receptors (GPCRs) are the largest family of cell-surface signaling proteins. They are receptors for an extraordinary range of structurally diverse agonists in the extracellular fluid, including endogenous hormones, neurotransmitters, and paracrine regulators, as well as multiple exogenous ligands (1, 2). Due to their critical importance in the control of most patho-physiological processes, GPCRs are the primary target for over 30% of the clinically used drugs (3, 4). The established mechanism of GPCR activation is that agonist binding results in conformational changes in the receptor that activate the Gα subunits of heterotrimeric G proteins, leading to the dissociation of Gβγ dimers from Gα. Activated Gα and Gβγ then initiate downstream signaling processes (5). To control the duration and magnitude of this signaling, activated receptors are phosphorylated by G protein-coupled receptor kinases (GRKs) or other kinases, and then interact with β-arrestins, which mediate receptor desensitization and endocytosis (6). Depending on the receptor and the agonist, internalized receptors are then sorted to lysosomes for degradation, or move to the plasma membrane for another cycle of activation (7, 8). However, a common feature of GPCRs is that a single receptor can interact with multiple endogenous and exogenous ligands, each of which may activate the receptor in different ways. For example, a large number of endogenous opioid neuropeptides as well as many different opiate drugs interact with opioid receptors, and different opioids and opiates result in divergent processes of receptor activation and regulation (9). Thus, the simplistic view of receptor activation and regulation has been revised by the appreciation that different agonists of the same receptor can result in distinct patterns of signaling and regulation.

The early two-state model of receptor function suggested that a receptor adopts active conformation upon ligand binding. This model considered only one active state, leading to a single functional readout. However, increased understanding of receptor signaling has revealed that different ligands can initiate distinct signaling events through the same GPCR. The heterogeneity of signaling events by a single GPCR can include different maximum responses from a single pathway (i.e., full or partial agonism) or activation of distinctly different signaling pathways by different agonists. The capacity of different agonists to initiate signaling of the same GPCR by distinct mechanisms is referred to as biased agonism or signaling (10, 11), and has been described for many GPCRs, including opioid receptors (12), angiotensin receptors (13), and glutamate receptors (14). This phenomenon of signaling bias is not surprising because GPCRs are flexible proteins that interact with multiple ligands and regulatory proteins, all of which may influence the capacity of the receptor to signal by particular mechanisms. Indeed, recent advances in our understanding of the structure of GPCRs in various activation states has revealed that a single GPCR can exist in multiple active conformations that may favor coupling to different signaling pathways (15, 16).

This review focuses on the capacity of different proteases and synthetic ligands to induce biased signaling of protease-activated receptors (PARs). The PARs are a family of four GPCRs (PAR_1–4_) that belong to group A rhodopsin-like GPCR subfamily. The first family member, PAR_1_, was identified as a receptor for thrombin, a serine protease coagulation factor (17). PAR_2_ was subsequently identified as a receptor for the serine protease trypsin (18), followed by PAR_3_, another thrombin receptor (19), and PAR_4_, a receptor for both thrombin and trypsin (20). PARs are expressed in many tissues and cell types, where they regulate multiple patho-physiological processes, including hemostasis, inflammation, pain, cellular proliferation, and healing (21–23). However, in addition to thrombin and trypsin, a large number of proteases have been identified that can cleave PARs. In some cases, these proteases cleave at the same sites as thrombin or trypsin and thereby initiate common signaling events. However, in other cases, proteases cleave PARs at distinct sites, and either activate distinct signals (biased agonism), or disarm the receptor by removing or destroying tethered ligand domains (receptor antagonism). We will review mechanisms by which various proteases and synthetic agonists activate PARs, and will discuss the implications of protease-biased signaling of PARs for patho-physiological control and therapeutic targeting.

## Mechanisms of Canonical Activation and Signaling of PARs

**Figure 1:**
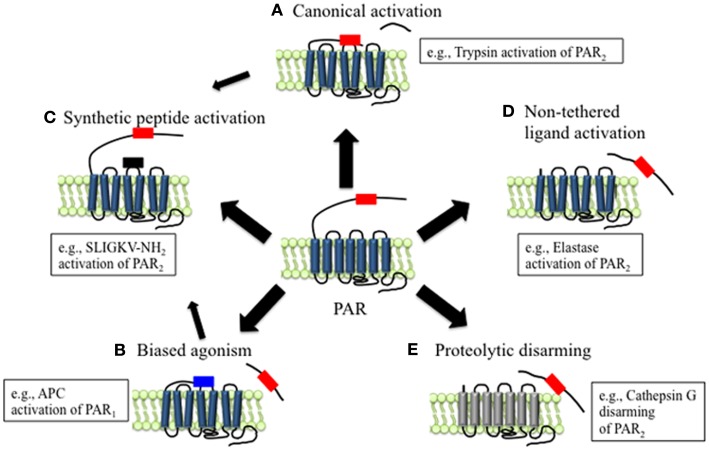
**Mechanisms of canonical and biased PAR signaling**. **(A)** Canonical mechanisms of PAR signaling. Proteases such as trypsin and thrombin cleave PARs at canonical cleavage sites, unmasking the tethered ligand domain, which binds to the second extracellular loops of the cleaved receptors. PARs that are activated by such mechanisms often couple to multiple G protein-dependent and β-arrestin-dependent signaling pathways. **(B)** Biased mechanisms of PAR signaling. Proteases such as elastase, MMP1, and APC cleave PARs at sites distinct from the canonical cleavage site. Cleavage may unmask a new tethered ligand that could interact with domains in the cleaved receptor, leading to the activation of unique and biased signaling pathways. **(C)** APs are synthetic peptides that mimic the tethered ligands revealed by proteases that cleave at canonical or biased sites. APs can activate the same pathways as proteases, although tethered ligand and soluble peptides may also trigger different signaling pathways and generate biased signal. **(D)** Some proteases such as elastase that cleave PARs to not appear to reveal tethered ligands, suggesting that proteolysis alone may activate the receptor. **(E)** Proteolytic disarming of PARs. Proteases such as cathepsin G cleave PARs and remove or destroy tethered ligands, thereby disarming proteolytic activation.

Unlike other GPCRs, the endogenous ligands for PARs reside within the extracellular N-terminus of the receptors. Receptor cleavage at the defined sites within the N-terminus by proteases such as thrombin and trypsin reveals these tethered ligands that, once exposed, can bind to regions in the second extracellular loops of the cleaved receptors, initiating conformational changes in the receptors that activate downstream signals (23). This is the canonical mechanism of PAR activation (Figure 1A). There are subtle differences in the mechanisms by which different proteases initiate the canonical pathways of receptor activation, which depend on the protease and PAR in question. For example, thrombin first binds to PAR_1_ and PAR_3_; this action facilitates receptor cleavage and exposure of the tethered ligand sequence. Mutation of the binding site reduces the efficacy with which thrombin activates these receptors, and mutation of the cleavage site prevents receptor activation (17, 19). On the other hand, trypsin activates PAR_2_ directly, without first binding to the receptor (18, 24). Accessory proteins can also influence the capacity of proteases to activate PARs. In particular, proteins that anchor proteases to the plasma membrane can enhance proteolytic activation. For example, during tissue damage and inflammation, tissue factor (TF) binds coagulation factors (F) VIIa, which in turn activates FX to FXa. FXa and its co-factor FVa promote conversion of prothrombin to thrombin, and subsequent PAR_1_ activation (25). Besides promoting thrombin activation, FVIIa and FXa both can signal directly through PAR_1_ and PAR_2_, although the efficiency and potency of receptor activation is substantially enhanced when they are coupled with TF (26). Similarly, the proteolytic activity of the anticoagulant activated protein C (APC) toward PARs is largely regulated by its association with the endothelial protein C receptor (EPCR) at the surface of endothelial cells (27, 28).

Support for the tethered ligand mechanism of PAR activation is provided by the observation that synthetic peptides, referred to as activating peptides (APs), that mimic the tethered ligand domain can also activate certain PARs directly, without the requirement for proteolysis (Figure 1C). Peptides mimicking the tethered ligands of PAR_1_, PAR_2_, and PAR_4_ can directly activate these receptors, although with a considerably lower potency than the activating proteases, especially in the case of PAR_4_ (17, 18, 20). The higher EC_50_ values of APs compare to those of proteases possibly reflect the differences between a tethered ligand and an untethered ligand in solution. PAR_3_ is not activated by tethered ligand-derived peptides, and appears to be unable to signal directly, but rather to serve as a co-factor for other PARs, such as PAR_1_ and PAR_4_ (29, 30).

Activating peptides have been considered to mimic the effects of proteases and have been widely used to probe the functions of PARs without the use of proteases, which can cleave multiple other proteins that may influence outcomes. However, this is not always the case because in some circumstances proteases and APs agonists can exert different effects. For example, in human brain microvascular endothelial cells, thrombin activation of PAR_1_ triggers endothelial barrier permeability, whereas PAR1-AP (SFLLRN-NH_2_) has no significant effect (31). In addition, the signaling properties of a PAR_2_ mutant with substitutions within the trypsin-revealed tethered ligand domain differ from those of APs with the same substitutions, suggesting distinct activation modes by tethered versus soluble peptides (32). The divergent signaling effects of proteases and APs provide evidence for biased signaling of PARs.

## Tissue-Specific Complexity and Diversity of PAR Activation and Signaling

In addition to the diversity of signals that can originate from the same receptor after activation by proteases or synthetic agonists (i.e., biased signaling), many other factors also affect patho-physiological outcome of PAR activation. These factors include the availability of activated proteases as well as the existence of regulatory and accessory proteins in different tissues and cell types.

The availability of active, functional proteases is a key requirement of PAR signaling, and the predominant endogenous proteases that activate PARs may vary in different patho-physiological states. For instance, the compliment of available active proteases varies markedly during the course of inflammation and healing, depending on the presence of immune cells, which are the source of many proteases, and on the existence of endogenous inhibitors. The compliment of proteases of mast cells, neutrophils, eosinophil, and macrophages, which participate in different phases of inflammation, varies considerably. For example, as the first responders to microbial infection, neutrophil produce elastase, cathepsin G, and proteinase-3 (33), whereas macrophages, which mediate chronic inflammation, release plasmin, matrix metalloproteinases (MMPs), and cathepsin S (34).

Although PARs can be activated by distinct proteases under different conditions, proteases that cleave PARs at the same sites would be expected to activate the same canonical signaling pathway and to induce common patho-physiological outcomes. However, the consequences of PAR cleavage could vary considerably if the activated proteases cleave PARs at distinct sites and are biased agonists or even antagonists, as discussed below. Indeed, at any one time multiple proteases would likely be activated and capable of cleaving PARs at distinct sites with unique outcomes. Thus, the active conformation of PARs may vary depending on the milieu of available proteases, which may differ between health and disease conditions. For example, during the initial stages of inflammatory processes such as inflammatory bowel diseases or chronic obstructive pulmonary disease, infiltration of neutrophils leads to increased level of elastase (35, 36), a biased agonist of both PAR_1_, and PAR_2_ (37, 38) (discussed below). Further complexity is provided by the presence of endogenous protease inhibitors that control the activity of proteases (39).

The outcome of PAR activation by the same protease or synthetic agonist can also vary between tissues and cell types. For example, thrombin and PAR_1_-AP cause relaxation of the intact coronary artery but contraction when the endothelium is removed, indicating distinct outcomes of PAR_1_ activation in endothelial versus vascular smooth muscle cells, possibility due to formation of different signaling complexes (40).

## Mechanisms of Biased Activation and Signaling of PARs

Compared to other GPCRs, the N-terminal domains of PARs are particularly susceptible to proteolysis. Although the reasons for this susceptibility are not fully understood, they probably relate to the presence of protease binding sites on the receptors, the existence of multiple scissile bonds, and the lack of groups that would sterically hinder proteolysis. However, the outcome of PAR activation depends on the site of proteolytic cleavage. Those proteases that cleave PARs at the conserved activating sites reveal tethered ligands that trigger the canonical signaling pathways (Figure 1A). Proteases that cleave PARs at distinct sites can act as biased agonists by triggering signals that are distinct from those activated by the canonical pathways (Figures 1B,D). In some cases, these alternative signaling mechanisms appear to involve exposure of distinct tethered ligands (Figure 1B). However, in other instances receptor cleavage *per se* may generate a conformational change that is sufficient to activate the receptor (Figure 1D). Alternatively, proteases can destroy or remove tethered ligand domains, forming N-terminally truncated receptors that are unresponsive to further activation by other proteases (Figure 1E).

Activated PARs can couple to multiple G protein-dependent (Gαq, Gα12/13, Gαi, Gαs, and Gβγ) and β-arrestin-dependent pathways. Although in many instances a particular protease or synthetic agonist can activate more than one of these pathways, in some cases proteases and synthetic agonists activate a single pathway. By comparing and categorizing the signaling pathways that are initiated by different proteases and synthetic agonists with the overall outcome of receptor activation, it is possible to identify the primary signaling pathways responsible for PAR-mediated patho-physiological responses (Tables 1–3). Moreover, a comprehensive understanding of the mechanisms and outcomes of PAR signaling by different proteases and synthetic agonists can guide the development of agonists and antagonists that may selectively activate or inhibit disease-relevant pathways. This approach has implications for development of pathway-specific therapies.

**Table 1:** **Activation of PAR_1_ by different proteases, their cleavage sites, synthetic activating peptide sequence, signaling pathways, and physiological effects**.

Receptor		Protease	Cleavage site	Activating peptide	Signaling pathways	Physiological response	Reference
**PAR_1_**		Thrombin				Platelet aggregation, endothelial barrier disruption, vascular smooth muscle cells proliferation	(17, 41, 42)
	Canonical cleavage	Factor Xa	^38^LDPR ↓SFLL^45^	SFLLRN-NH_2_	Gαq/Ca^2+^, Gα12/13-Rho, β-arrestin/ERK1/2	Pro-inflammation, endothelial barrier protection, inhibition of cancer cell migration, fibroblast proliferation	(43 –45)
		Plasmin				Platelet activation and deactivation (by non-specific cleavage)	(46)
		MMP1	^36^ATLD ↓PRSF^43^	PRSFLLRN-NH_2_	Gα12/13-Rho, MAPK	Platelet thrombogenesis and clot retraction, disruption of barrier function, matrix remodeling, vascular angiogenesis	(47 –49)
		MMP13	^39^DPRS ↓FLLR^46^	Not studied	Gαq/Ca^2+^, ERK1/2	Participate in β-AR over activation-dependent cardiac dysfunction	(50)
	Non-canonical cleavage	Elastase	^42^SFLL ↓RNPN^49^	RNPNDKYEPF-NH_2_	Gαi/MAPK	Stress fiber formation and endothelial barrier permeability	(37)
		APC	^43^FLLR ↓NPND^50^	NPNDKYEPF-NH_2_	β-arrestin/Rac1, Akt	Cytoprotective, endothelial barrier protection	(51 –54)
		Proteinase-3	^33^ATNA ↓TLDP^40^	TLDPRSF-NH_2_	Gαi/MAPK	Stress fiber formation and endothelial barrier permeability	(37)
	Other proteases	Granzyme K	N.D.	N.D.	ERK1/2, p38 MAPK	Cytokine secretion and fibroblast proliferation	(55)

## PAR_1_ Activation and Signaling

### Canonical activation of PAR_1_

**Figure 2:**
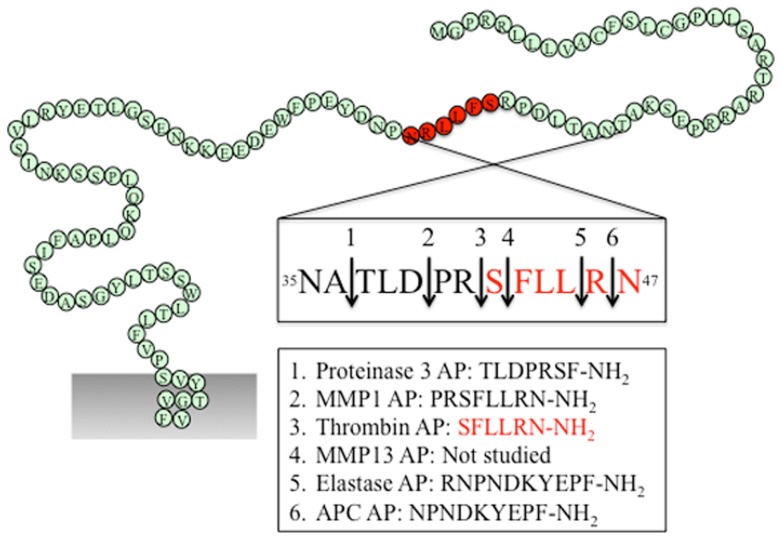
**PAR_1_ N-terminus with major cleavage sites identified**. N-terminus of human PAR_1_ (1–114). The residues in red denote the canonical tethered ligand and a corresponding AP that is revealed by thrombin cleavage. The cleavage sites for different proteases and the corresponding AP for each protease are indicated in the boxes. Gray shading represents membrane.

As the first identified PAR, the canonical mechanisms of PAR_1_ activation and signaling have been extensively investigated. An interaction between thrombin’s anion-binding exosite I and a negatively charged region in the extracellular N-terminus of PAR_1_ (^51^DKYEPF^56^) increases the affinity of thrombin for the receptor and facilitates cleavage (17). Binding of thrombin enables the enzyme to cleave the receptor at position R^41^/S^42^, which reveals the tethered ligand domain beginning with SFLLRN in human PAR_1_, and initiates downstream signaling cascades (Figure 2). After cleaving PAR_1_, thrombin may remain associated with the receptor to facilitate its action on other thrombin receptors, such as PAR_4_ (83). Thrombin-activated PAR_1_ can trigger multiple G protein-dependent and -independent signaling pathways, including Gαq, Gαi, and Gα12/13. A region spanning the thrombin cleavage sites act as a “hot spot” for many proteases, including granzyme A, plasmin, and FXa, that cleave at the same site as thrombin and trigger similar cellular responses (Table 1). Proteases that cleave at other sites can induce biased signaling of PAR_1_.

### Biased activation of PAR_1_

Several proteases have been identified that cleave PAR_1_ at sites different from the canonical thrombin site, leading to distinct patho-physiological outcomes.

#### Activated protein C

Activated protein C is a natural anticoagulant with powerful anti-inflammatory and cytoprotective activities (84). In many cases, APC exerts its protective effect *via* EPCR and PARs. On the surface of endothelial cells, binding of protein C to EPCR promotes its activation by thrombin, and EPCR-bound APC in turn exerts its cytoprotective effect by cleaving and activating PAR_1_ (51). Different from thrombin-mediated PAR_1_ activation, APC activation of PAR_1_ requires colocalization of PAR_1_ with EPCR in caveolae microdomains in the form of a signaling complex with caveolin-1 (85, 86). Besides subcellular localization, the differential PAR_1_-dependent cellular responses induced by thrombin and APC may also be explained by their distinct cleavage sites. APC cleaves PAR_1_ at the canonical cleavage site R^41^/S^42^, as well as at an alternate site R^46^/N^47^, with the latter being the primary cleavage site that is responsible for its cytoprotective effect (51, 52) (Figure 2). A synthetic AP corresponding to the tethered ligand that would be revealed by this alternate cleavage (N^47^PNDKYEPFWEDEEKNESGL^66^-NH_2_) mimics the protective effects of APC both *in vitro* and *in vivo*. Cleavage of PAR_1_ at R^46^/N^47^ by APC leads to β-arrestin 2-mediated Rac1-activation independent of G protein (53). Both APC and its AP stimulate PAR_1_-dependent phosphorylation of glycogen synthase kinase 3 β and Akt (51). In contrast to thrombin-activated PAR_1_, APC-cleaved PAR_1_ fails to activate extracellular signal-regulated kinase (ERK)1/2. Thus, APC and thrombin cleave PAR_1_ at different sites leading to the exposure of distinct tethered ligand agonists that activate different signaling pathways.

#### Matrix metalloproteinases

Matrix metalloproteinases are a family of 28 zinc-dependent proteases that play important roles in regulating platelet and endothelial function (87). Two human MMPs, MMP1 and MMP13, and one murine MMP, MMP1a, exhibit activity toward PAR_1_. Both MMP1 and MMP13 cleave PAR_1_ at non-canonical sites (D^39^/P^40^ for MMP1, S^42^/F^43^ for MMP13, Figure 2), which either generate an extended tethered ligand with two more amino acids or a truncated tethered ligand lacking the first serine residue compared to the tethered ligand exposed by thrombin (Figure 2). Similar to thrombin, MMP1-cleaved PAR_1_ activates the Gα12/13-Rho-GTPase pathway, and also leads to mitogen-activated protein kinase (MAPK) signaling and platelet shape changes (47). However, MMP1-activated PAR_1_ is a weak agonist of Ca^2+^ signaling and platelet aggregation (47, 48). The biased cellular response between thrombin- and MMP1-activated PAR_1_ has also been studied in vascular smooth muscle cells. Thrombin activation of PAR_1_ leads to a supercontractile, differentiated phenotype that is pertussis toxin-sensitive, suggesting the involvement of Gαi activation, whereas MMP1 activation of PAR_1_ results in a dedifferentiated phenotype *via* a Gαi-independent mechanism (49). These differences in signaling in vascular smooth muscle cells may account for the opposite effects of thrombin and MMP1 on the development of arterial stenosis following arterial injury (49).

Whereas MMP1 is mostly expressed in vascular endothelial cells, platelets, and macrophages, MMP13 is prominently expressed in cardiac fibroblasts and cardiomyocytes. Expression of MMP13 is increased in cardiac fibroblasts after β_2_-adrenergic receptor activation (50). MMP13 cleaves PAR_1_ one amino acid downstream from the thrombin site at S^42^/F^43^. In ventricular myocytes of neonatal rats, MMP13-activated PAR_1_ leads to phosphorylation of ERK1/2 and p38 MAPK. However, when compared to thrombin, MMP13 elicits similar levels of ERK1/2 activation but only modestly stimulates inositol phosphate formation (50). Due to the close proximity of the thrombin and MMP13 cleavage sites, it is likely that MMP13 activates PAR_1_ by a tethered ligand mechanism. Whether this single amino acid difference in the tethered ligands is sufficient to generate biased signaling of PAR_1_ remains to be determined.

#### Neutrophil proteases

During acute inflammation, neutrophils are the first cells infiltrate to the inflammatory site and are important mediators of inflammatory response. Elastase and proteinase-3 are stored in large quantities within secretory granules and are activated and released into the extracellular environment during inflammation (88). Recent studies show that both proteases are biased agonists for PAR_1_ (37). Elastase cleaves PAR_1_ at L^45^/R^46^, and proteinase-3 cleaves PAR_1_ at A^36^/T^37^ (Figure 2). Similar to thrombin, elastase and proteinase-3 activate PAR_1_ via tethered ligand mechanism. In contrast to thrombin-cleaved PAR_1_, which activates Gα12/13- as well as Gαq-mediated signaling pathways, elastase, proteinase-3 and their corresponding APs (elastase-AP: RNPNDKYEPF-NH_2_; proteinase-3-AP: TLDPRSF-NH_2_) induce Gαi-mediated MAPK activation, regardless of their distinct cleavage positions. Although proteinase-3 cleaves prior to canonical activation site (five amino acids N-terminal to the thrombin cleavage site), proteinase-3 fails to induce Ca^2+^ signaling, suggesting the possibility that the extra 5 residues (TLDPR) may has an inhibitory role in coupling activated PAR_1_ to Gαq and Ca^2+^ mobilization (37).

### PAR_1_ activation by synthetic ligands

Several synthetic APs corresponding to the tethered ligands exposed by proteolytic activation of PAR_1_ have been evaluated *in vitro* or *in vivo*. These include AP corresponding to tethered ligands revealed by thrombin (SFLLRN-NH_2_), neutrophil elastase (RNPNDKYEPF-NH_2_), neutrophil proteinase-3 (TLDPRSF-NH_2_), MMP1 (PRSFLLRN-NH_2_), and APC (NPNDKYEPF-NH_2_) (Figure 2). Since these proteases cleave PAR_1_ between residues 35 and 45, the APs share considerable homology. For example, the thrombin and MMP1 APs differ by only two amino acids, whereas the APC AP is only one amino acid shorter than the elastase-AP. However, regardless of their sequence homology, different APs display considerable signaling bias. For instance, the thrombin AP SFLLRN-NH_2_ activates Gαq-mediated signaling (89), whereas the MMP1 AP PRSFLLRN-NH_2_ is a weak agonist of Gαq signaling and preferentially activates the Gα12/13 pathway (47). In addition, elastase-AP RNPNDKYEPF-NH_2_ stimulates ERK1/2 phosphorylation *via* Gαi but the APC AP NPNDKYEPF-NH_2_ activates ERK by a β-arrestin-dependent but G protein-independent mechanism (37, 51).

The differences in AP-induced signals lead to distinct physiological outcomes. For example, in human umbilical vein endothelial cells, elastase-AP and APC-AP suppress thrombin-stimulated increase in endothelial barrier permeability, whereas proteinase-3-AP and MMP1-AP have the opposite effect (37).

Other structurally distinct synthetic peptides can also activate PAR_1_. YFLLRNP-NH_2_, a peptide that differs from the thrombin AP by a single amino acid, is able to cause platelet shape changes by a Ca^2+^-independent mechanism (90) that may involve Gα12/13-dependent activation of Rho kinase (91). TFRRRL-NH_2_, a peptide derived from the C-terminus of P2Y receptor, can activate PAR_1_ on human platelets and stimulate Gα12/13- and Gαq-dependent changes in platelet shape and aggregation (92).

In addition to providing evidence for the capacity of PARs to exhibit signaling bias, studies of APs signaling have also provided insights into the molecular mechanisms of PAR_1_ activation. Thus, the thrombin AP SFLLRN-NH_2_ is able to activate both Gαq and Gα12/13 pathways, with a preference for Gαq, whereas the MMP1 AP PRSFLLRN and YFLLRNP activate only Gα12/13 signals (47). These observations suggest the importance of the serine residue at position 1 of the peptide for Gαq activation, whereas either replacing it with another residue or extending the peptide with additional N-terminal residues both result in a reduction in Gαq activation. The precise mechanisms of how these peptides interact with cleaved PAR_1_ to induce these divergent signals remains to be determined.

## PAR_2_ Activation and Signaling

### Canonical activation of PAR_2_

**Figure 3:**
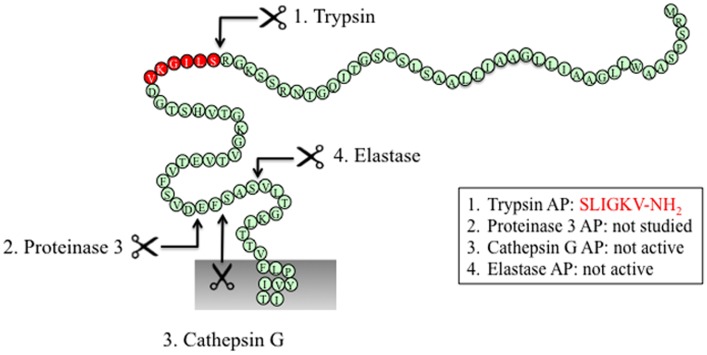
**PAR_2_ N-terminus with major cleavage sites identified**. N-terminus of human PAR_2_ (1–84). The residues in red denote the canonical tethered ligand and a corresponding AP that is revealed by trypsin cleavage. The corresponding APs for each protease are indicated in the boxes. Gray shading represents membrane.

The canonical mechanism of activation of PAR_2_ by trypsin involves hydrolysis at position R^36^/S^37^, which reveals the tethered ligand SLIGKV (human) (24) or SLIGRL (mouse) (18) (Figure 3). This exposed tethered ligand then interacts with the second extracellular domain of the cleaved receptor and trigger multiple G protein-dependent and -independent signaling pathways (93). Trypsin-activated PAR_2_ leads to the activation of Gαq-mediated Ca^2+^ mobilization (94), Gαs-dependent formation of cAMP (95), Gα12/13-mediated increasing in Rho-Kinase activity (95), recruitment of β-arrestin-1 and -2 (96), ERK1/2 phosphorylation (97, 98), and subsequent receptor internalization and degradation (94, 99).

Several other proteases cleave PAR_2_ at the canonical site (Table 2). Serine proteases that activate canonical PAR_2_ signaling include trypsin I/II (18, 94), trypsin IV (100, 101), tryptase (59, 102), coagulation factors VIIa and Xa (26), acrosin (103), granzyme A (104), and kallikrein 2, 4, 6, and 14 (63, 64, 105). Proteases that cleave PAR_2_ at the canonical activation site would be expected to reveal the conserved tethered ligand domain and to activate the same compliment of signaling pathways as trypsin. Despite the fact that these proteases cleave PAR_2_ at the same site as trypsin, the potency with which they activate PAR_2_ shows considerable variability. This variability may be due to different rate and efficiency of cleavage between different proteases. Although not all the K_cat_ values for proteases cleaving PAR_2_ have been reported, published *in vitro* peptide proteolytic assays show marked differences in the kinetics of cleavage. Cleavage at sites that disable the receptor may also contribute to the variable potency of proteases-mediated signaling. Compared to trypsin, tryptase is a partial agonist of PAR_2_-dependent Ca^2+^ signaling, which may be related to a second cleavage site at R^41^/S^42^, which would deactivates the receptor and limit its potential to induce further Ca^2+^ signals (102). Post-translational modification of PARs may also affect their susceptibility to proteolytic activation. Although mast cell tryptase can activate PAR_2_, its ability to do so is limited by receptor glycosylation, which presumably sterically hinders hydrolysis at R^36^/S^37^ (106). Other proteases such as kallikrein-related peptidase 14 and gingipain-R have been shown to signal by PAR_2_-dependent mechanism, although the cleavage sites need to be confirmed (70, 71).

**Table 2:** **Activation of PAR_2_ by different proteases, their cleavage sites, synthetic activating peptide sequence, signaling pathways, and physiological effects**.

Receptor		Protease	Cleavage site	Activating peptide	Signaling pathways	Physiological response	Reference
**PAR_2_**		Trypsin				Pro-inflammation, induction of hypotension, mechanical and thermal hyperalgesia, cardio protective (reduced infarct size)	(42, 56 –58)
	Canonical cleavage	Tryptase	^33^SKGR ↓SLIG^40^	SLIGKV-NH_2_	Gαq/Ca^2+^, Gα12/13-Rho, MAPK ERK1/2, β-arrestin, Akt, Gαi and Gαs/cAMP	Pro-inflammatory and hyperalgesia; increase paracellular permeability of intestine; mast cell degranulation; cell proliferation	(59–61)
		Factor VIIa				Cancer cells migration and invasion	(62)
		Factor Xa				Cancer cells migration and invasion	(62)
		KLKs				Cell proliferation	(63, 64)
		Elastase	^64^FSAS ↓VLTG^71^	Not active	Rho/ERK1/2	N.D.	(38, 65)
	Non-canonical cleavage	Proteinase-3	^57^VFSV ↓DEFS^64^	Not active	N.D.	N.D.	(38)
		Cathepsin G	^61^VDEF ↓SASV^68^	Not active	N.D.	N.D.	(38, 65)
		Cathepsin S	^53^VTVE ↓TVFS^60^	TVFSVDEFSA-NH_2_	Gαs/cAMP	Pro-inflammatory, visceral hyperalgesia, itch	(66 –69)
	Other proteases	Gingipain-R	N.D.	N.D.	Gαq/Ca^2+^, ERK1/2	Activate human gingival fibroblasts and modulate immune response	(70)
		KLK 14	N.D.	N.D.	Gαq/Ca^2+^, ERK1/2	Colon tumorigenesis, pro-inflammatory	(71)

**Table 3:** **Activation of PAR_3_ and PAR_4_ by different proteases, their cleavage sites, synthetic activating peptide sequence, signaling pathways, and physiological effects**.

Receptor		Protease	Cleavage site	Activating peptide	Signaling pathways	Physiological response	Reference
**PAR_3_**	Canonical cleavage	Thrombin	^35^LPIK ↓TFRG^42^	TFRGAP-NH_2_ (for PAR1 and PAR2)	ERK1/2	IL-8 production	(19, 72, 73)
	Non-canonical cleavage	APC	^38^KTFR ↓GAPP^45^	GAPPNSFEEFPFS	N.D.	Cytoprotective	(27, 74)
**PAR_4_**	Canonical cleavage	Thrombin				Platelet activation and aggregation; platelet endostatin release	(75–77)
		Trypsin	^44^PAPR ↓GYPG^51^	GYPGQV-NH_2_	Ca^2+^	Neutrophil recruitment	(78)
		Plasmin				Platelet activation and aggregation	(79, 80)
		Cathepsin G				Platelet activation and aggregation	(81)
	Other proteases	MASP1	N.D.	N.D.	Ca^2+^ NF-kB, p38 MAPK	N.D.	(82)

### Biased activation of PAR_2_

As is the case for PAR_1_, several proteases have been identified that cleave PAR_2_ at distinct sites, leading to signaling bias.

#### Neutrophil proteases

Neutrophil elastase, proteinase-3, and cathepsin G can all cleave PAR_2_. Neutrophil elastase and cathepsin G were first considered to be deactivating proteases due to their ability to cleave downstream from the canonical trypsin site and thereby disarm PAR_2_ and attenuate trypsin-dependent Ca^2+^ signals (104). However, a recent study suggests that these cleavage events may also induce PAR_2_ signaling bias.

Elastase cleaves PAR_2_ between S^68^/V^69^ (38) (Figure 3). Treatment of KNRK-PAR_2_ cells with elastase does not induce Ca^2+^ signals but does trigger PAR_2_-dependent ERK phosphorylation by a pathway that involves Gα12/13-mediated activation of Rho kinase. In contrast to trypsin, elastase does not trigger β-arrestin recruitment or receptor internalization. A synthetic peptide corresponding to a tethered ligand domain that would be revealed by elastase (VLTGKLTTVFL-NH_2_) fails to mimic the action of elastase and to activate ERK or to stimulate Ca^2+^ signals in KNRK-PAR_2_ cells, suggesting that elastase could activate PAR_2_ by a mechanism that does not require tethered ligand binding to the cleaved receptor. Indeed, the elastase cleavage site is close to the first transmembrane domain of PAR_2_, which would suggest that a tethered ligand mechanism is unlikely. Presumably, cleavage *per se* may allow PAR_2_ to adopt an active conformation that favors activation of certain signaling pathways. Although the functional relevance of elastase activation of PAR_2_ is uncertain, it may contribute to inflammatory diseases in which this protease-receptor pair is involved. For example, neutrophil elastase activity is elevated in patients with ulcerative colitis as well as dextran sulfate sodium-induced colitis in mice (107), and the elastase inhibitor serpin B1 (107) and PAR_2_ antagonism and deletion protect against colitis (108). Therefore, it will be important to determine whether elastase triggers PAR_2_-dependent inflammatory signaling in colonocytes or immune cells that express PAR_2_.

Similar to elastase, neutrophil cathepsin G and proteinase-3 both cleave PAR_2_ downstream of the canonical trypsin site (cathepsin G: P^65^/S^66^ and proteinase-3 V^62^/D^63^) (38) (Figure 3). However, neither cathepsin G nor proteinase-3 stimulate PAR_2_-dependent Ca^2+^ signals or activate ERK phosphorylation or receptor internalization (38). Although these proteases can disarm PAR_2_ by removing the trypsin-exposed tethered ligand, it remains to be determined whether they also induce biased signaling or they act as antagonists for the receptor. The functional relevance of PAR_2_ cleavage by cathepsin G and proteinase-3 is unknown.

#### Cathepsin S

Cathepsin S, a lysosome cysteine protease of the papain family, is expressed by antigen-presenting cells, including macrophages, microglial cells, B-lymphocytes, and dendritic cells, and contributes to antigen presentation and adaptive immunity (109). Inflammatory mediators promote cathepsin S secretion from macrophages and microglial cells (66, 110), and there is increased cathepsin S activity in inflamed tissues, including synovial fluid from patients with rheumatoid arthritis (111) and colonic secretions from mice with colitis (67). Cathepsin S is active at both lysosomal acidic pH and extracellular pH (66), and may thus be able to activate PARs. When administered into the colonic lumen of wild-type mice to replicate the increased luminal cathepsin S detected in mice with colitis, cathepsin S causes visceral pain (67). This hyperalgesia is absent from PAR_2_ knockout mice, suggesting that cathepsin S can activate PAR_2_. Current studies are investigating whether cathepsin S can activate biased PAR_2_ signaling and to determine the functional relevance of this process for PAR_2_-mediated inflammation and pain.

### PAR_2_ activation by synthetic ligands

In addition to proteases, synthetic peptides that mimic the trypsin-exposed tethered ligand can also activate PAR_2_. The hexapeptides SLIGRL-NH_2_ and SLIGKV-NH_2_ corresponding respectively to the mouse and human trypsin-revealed tethered ligands for PAR_2_ have been extensively used as tools to study PAR_2_ function despite their relatively low potency. Both peptides have been shown to induce a Gαq-dependent increase in [Ca^2+^]_i_ (26, 32, 94, 104, 112–114), ERK1/2 activation (62, 98, 115), as well as β-arrestin recruitment and subsequent internalization (98, 112). The effect of SLIGRL-NH_2_ on cellular cAMP levels is controversial. In rabbit smooth muscle cells, SLIGRL-NH_2_ decreases forskolin-induced accumulation of cAMP in a pertussis toxin-sensitive manner (114). On the other hand, in HEK293 cells and human keratinocytes, SLIGRL-NH_2_ increases cAMP formation (95, 116). In keratinocytes, SLIGRL-NH_2_ stimulates a pertussis toxin-insensitive and cAMP/PKA-independent activation of Rho kinase (95). Thus, activated PAR_2_ may trigger different G protein pathways depending on the cellular context and the availability of other components of the signaling complex.

Although these APs can cause robust PAR_2_ signaling, mutagenesis studies have highlighted that different residues within the activating ligand domain may determine the preference of the receptor to activate certain signaling pathways such as Ca^2+^ versus MAPK. These studies not only support the idea that PAR_2_ can initiate biased signaling, they also provide important information on the mechanisms of PAR_2_ activation. Mutagenesis of rat PAR_2_ has revealed that the first two residues (S^37^L^38^) from the AP are critical for PAR_2_ activation. Both mutated PAR_2_ receptor with substitution of these two residues to alanine and its corresponding soluble peptide ligand (AAIGRL-NH_2_) exhibit little or no activity (117). Notably, a subsequent study found that another analog peptide of SLIGRL-NH_2_, SLAAAA-NH_2_, also showed minimum Ca^2+^ signaling activity but was able to induce ERK1/2 phosphorylation via a Rho-kinase-dependent mechanism (32). Although the PAR_2_ binding pocket for APs has yet to be fully identified, the importance of extracellular loop 2 in PAR_2_ activation by soluble peptides has been studied, leading to the suggestion that the glutamic acid residues (E^232^E^233^ in rat PAR_2_, and E^232^Q^233^ in human PAR_2_) may interact with the basic arginine residue at position 5 of the AP. This residue is required for activity since mutation of either receptor or the peptide results in loss of Ca^2+^ signal (118). It will be important to examine whether distinct or overlapping clusters of residues of PAR_2_ are responsible for binding to different regions of the AP, thereby triggering different signaling events.

### PAR_2_ activation by small molecules

Recent advances in our understanding of the structure-activity relationships of various PAR_2_ ligands have facilitated the development of small molecule PAR_2_ agonists, albeit of limited potencies. The potential for these compounds to exhibit signaling bias has not been fully investigated, since most studies have only examined their ability to affect PAR_2_-dependent Ca^2+^ signals. AC-98170 is a partial agonist of PAR_2_-dependent Ca^2+^ signaling (30% efficacy of SLIGRL-NH_2_), but with a lower EC_50_ than AC-55541, another small molecule agonist for PAR_2_ (119). Whether these compounds are biased agonists of different PAR_2_ signaling pathways remains to be investigated.

Small molecule PAR_2_ antagonists have also been developed. One such compound, GB88, is a competitive antagonist for both trypsin- and AP-induced Ca^2+^ signaling, although GB88 can selectivity activate other PAR_2_-dependent pathways, including cAMP formation, Rho-kinase stimulation, and ERK1/2 phosphorylation (120). Thus, rather than acting as an antagonist for PAR_2_, GB88 may act as a biased agonist for this receptor. However, the antagonistic activity of GB88 is sufficient to attenuate PAR_2_-induced paw edema and acute inflammation, as well as collagen-induced arthritis in rats (121, 122), suggesting that relative contribution of PAR_2_ in these disease conditions might be primary *via* Ca^2+^-dependent pathways.

Like GB88, K-14585 also has a complex pharmacology. In human skin epithelial cells, K-14585 is able to block SLIGKV-NH_2_-induced inositol phosphate accumulation and p38 MAPK phosphorylation without affecting PAR_2_-mediated ERK1/2 activation (123). However, although not significant, K-14585 alone does induce a modest IP3 formation. Moreover, at a higher concentration, K-14585 triggers PAR_2_-dependent p38 MAP kinase phosphorylation (123). Taken together, K-14585 may actually act as a PAR_2_ agonist with relatively low potency.

## PAR_3_ Activation and Signaling

Although thrombin cleaves PAR_3_ at K^38^/T^39^, there is little evidence that the cleaved receptor is capable of signaling. Thrombin and a synthetic peptide corresponding to the putative tethered ligand failed to generate PAR_3_-dependent Ca^2+^ signals (19). However, this PAR_3_-derived AP is able to activate PAR_1_ and PAR_2_ (72). Instead of signaling in its own right, PAR_3_ appears to be a co-factor for the activation of other PARs, including PAR_4_ and PAR_1_ (27, 29, 30). Co-expression of PAR_3_ with PAR_4_ in COS-7 cells leads to over 10-fold increase in the efficiency of thrombin cleaving PAR_4_ compare to PAR_4_ expressed alone (EC_50_ from 0.3 to 0.05 nM) (29). However, with the appreciation of PAR biased signaling, further studies are warranted to examine the full repertoire of potential PAR_3_ signals.

In contrast to other PARs, fewer proteases have been identified that can cleave PAR_3_. Besides thrombin, APC is the only protease to be identified that exhibits proteolytic activity toward PAR_3_ (27, 74). In immortalized human and mouse podocytes, which have higher expression of PAR_2_ and PAR_3_ than PAR_1_ and PAR_4_, the maximum inhibitory effect of APC-dependent podocyte apoptosis requires cleavage of N-terminal domain of PAR_3_ by APC at the same position as thrombin (27). Cleavage of PAR_3_ by APC promotes dimerization between PAR_2_ and PAR_3_, and this is required for APC-dependent cytoprotective effect. A recent study reported a novel mechanism of APC activation of PAR_3_ *via* a cleavage at a non-canonical site (R^41^/G^42^ X instead of K^38^/T^39^) (74). Peptide hydrolysis experiments revealed a slow kinetics of APC cleavage of PAR_3_ (50% peptide cleavage reached after ~5 h), suggesting that other modulators may facilitate APC cleaving PAR_3_ in cells; indeed, the efficiency of this cleavage increased proportional to the expression of EPCR (74). This novel cleavage by APC generates a new tethered ligand domain starting with G^42^APPNS. Consistent with a tethered ligand activating mechanism, APC-AP (G^42^APPNSFEEFPFS^54^-NH_2_) is able to prevent thrombin-induced endothelial barrier disruption. Interestingly, an extended peptide generated from thrombin cleavage site (^40^TFGAPPNSFEEFPFS^54^-NH_2_) fails to do so. This suggests that APC and thrombin activation of PAR_3_ mediates different signaling profile, and thus is involved in different cellular response, respectively. However, similar to thrombin activation of PAR_3_, existence of another receptor, such as PAR_1_ is necessary for APC/PAR_3_-mediated signaling (74).

## PAR_4_ Activation and Signaling

PAR_4_ was identified by a homology search using amino acid query sequence derived from known sequences of PAR_1_, PAR_2_, and PAR_3_ (20). The putative protease cleavage site was identified (R^47^/G^48^), and the EC_50_ of thrombin toward PAR_4_ was much higher compare to other thrombin sensitive receptors PAR_1_ and PAR_3_ (5 nM for PAR_4_, and 0.2 nM for PAR_1_ and PAR_3_) (17, 19, 20). Further investigation suggests that this may be due to the lack of the thrombin binding site within the amino terminus of the receptor. Consistent with this observation, γ-thrombin, another isoform of thrombin that lacks a receptor binding site exhibits similar affinity on PAR_4_ compare to α-thrombin (20). Different from other PARs that can be cleaved preferentially by trypsin or thrombin, PAR_4_ exhibits similar sensitivity toward both enzymes. Both thrombin and trypsin activities toward PAR_4_ can be abolished by R^47^/A mutation of the receptor, suggesting that both enzymes cleave the receptor at the same site (20).

Compared to PAR_1_ and PAR_2_, little is known about biased signaling of PAR_4_, and to date no additional activating cleavage sites have been identified. However, although proteases such as plasmin can activate PAR_4_ by cleaving the receptor at the canonical site, thrombin and plasmin cleave PAR_4_ with different kinetics, possibility due to different mechanisms of action or the affinity of the proteases for the receptor (20). In mouse platelets, PAR_3_ binds to thrombin and thereby acts as a co-factor to facilitate thrombin cleavage and activation of PAR_4_ (29). However, in both transfected cell lines as well as platelet, instead of acting as a co-factor, the presence of PAR_3_ inhibits plasmin-mediated PAR_4_ activation leading to a decrease in intracellular Ca^2+^ mobilization and platelet aggregation (79). The mechanism that underlies these findings is not clear. However, since thrombin cleaves both PAR_3_ and PAR_4_ whereas plasmin can only cleave PAR_4_, the conformation of the PAR_3_–PAR_4_ receptor pair might be different after plasmin or thrombin cleavage. In addition, difference in binding affinities and kinetics of plasmin and thrombin for PAR_4_ may also result in distinct receptor-protease complex formation and lead to variation in downstream responses.

Cathepsin G is a neutrophil serine protease that plays an important role in inflammation. Cathepsin G can evoke PAR_4_-dependent Ca^2+^ signals in human platelets and in PAR_4_-transfected fibroblasts (81). Cathepsin G and PAR_4_ are upregulated in ulcerative colitis patients, and inhibition of cathepsin G and PAR_4_, but not PAR_1_ or PAR_2_, is protective (124). Although no direct evidence suggests that PAR_4_ is the target for cathepsin G in colitis, this study highlights the possibility of targeting cathepsin G or PAR_4_ as novel therapeutic approach.

Recently, mannose-binding lectin-associated serine protease-1 has been shown to cleave PAR_4_ but not PAR_1_ or PAR_2_ in endothelial cells, and to induce PAR_4_-dependent Ca^2+^ responses and activation of NF-κB and p38 MAPK pathways. However, the exact cleavage site remains to be determined (82).

## Signaling by PAR Dimers

Although most studies have examined signaling by monomeric PARs, considerable evidence suggests that PARs may form homo- or hetero-dimers and function as a complex. Dimerized PARs could adopt unique conformations and activate different signaling pathways compare to monomer (125).

As a receptor that exhibits little or no activity when expressed alone, PAR_3_ has been examined as a co-factor for other PARs. PAR_3_ can modulate the activity of PAR_1_ by potentiating its response to thrombin, thereby increasing endothelial barrier permeability without altering Ca^2+^ responses (30). This receptor dimer pair favors coupling to Gα13 over Gαq, whereas both pathways are similarly activated by PAR_1_ monomer (30). Thus, PAR_1_ may exhibit distinct signaling profiles in response to the same ligand when coupled to PAR_3_.

In mouse platelets, dimerization between PAR_3_ and PAR_4_ leads to negative regulation of PAR_4_-mediated Ca^2+^ mobilization and PKC activation without affecting Gα12/13 and Gαi activation, suggesting that PAR_4_ signaling is biased away from Gαq activation when coupled to PAR_3_ (126).

In human podocytes, APC cleavage of PAR_3_ leads to the formation of PAR_2_ and PAR_3_ heterodimers, which is essential for the anti-apoptotic actions of APC (127). Although the signaling pathways that regulate this activity remain to be defined, the observation that both PAR_2_ and PAR_3_ activating peptides were able to produce similar effects suggest that the formation of this hetero-dimer may stimulate signaling pathways that are similar to those activated by PAR_2_ monomers.

Another example of the contribution of receptor dimerization to biased PAR signaling is dimerization between PAR_1_ and PAR_2_. PAR_1_–PAR_2_ dimerization has been demonstrated in both overexpression system and endogenous expression system (128, 129). When PAR_1_ forms dimer with PAR_2_, the thrombin-revealed PAR_1_ tethered ligand can trans-activate PAR_2_ and trigger PAR_2_-dependent Gαi/Rac signaling, while PAR_1_-mediated Gαq and Gα12/13 signaling is switched off (130). In addition, recruitment of β-arrestin to the PAR_1_–PAR_2_ dimer exhibits distinct kinetic compared to each protomer, suggesting a potential alteration in β-arrestin-dependent ERK1/2 signaling (128). During the early development of sepsis, the effect of thrombin is vascular disruption whereas at the later phase of sepsis, with increasing expression of PAR_2_, thrombin induces a vascular protective effect that is mediated by PAR_2_/Rac1 activation (130).

Protease-activated receptors may act as co-factors or may dimerize either constitutively or in a ligand-dependent manner. The formation of dimers may play a role in organization of receptors at the cell surface, and may allosterically modulate the activation of either monomer or act as a complex that generates unique signaling outcomes.

## Regulation of PAR Signaling by Different G Protein Coupling

Different proteases can lead to biased PARs activation, and PAR signaling can also be modulated by co-factors and receptor dimerization. In addition, the interaction of PARs with different G proteins also has marked influence on the outcome of protease signals. In response to a single protease, PARs are able to couple to multiple G proteins. Although how this occur is not clear, recent studies using bioluminescence resonance energy transfer (BRET) approach suggest dynamic regulation of PAR_1_ and PAR_2_ coupling with multiple G proteins. In Cos-7 cells, both receptors spontaneously form pre-assembled complexes with Gαi, whereas they only couple to Gα12 following ligand stimulation, and with slow kinetics (131, 132). Further investigation revealed the existence of two different PAR populations that are responsible for coupling to different Gα protein. The existence of distinct receptor populations may be interpreted as receptor clustering in different membrane microdomains such as membrane raft and caveolin-containing vesicles (131). GPCRs located in lipid raft-enriched domains may assure certain conformations of the receptor that preferentially couple to specific G proteins compared to receptor located in non-raft membrane compartment (133). It would be of great interest to examine whether divergent PARs signaling in various cell types depends on the lipid component of the membrane and whether altering membrane lipid composition leads to a shift in PAR signaling profile, thereby contributing to bias signaling. This mechanism may also account for the tissue specificity of PAR signaling.

## Termination of PAR Biased Signaling

A growing number of proteases have been identified that can cleave PARs at distinct sites, leading to diverse signals. One striking observation is the capacity of different proteases to stimulate receptor endocytosis. It is well established that cleavage of PARs at canonical activation site leads to rapid receptor internalization in a β-arrestin-dependent (PAR_2_) or -independent (PAR_1_) manner (96, 134). Receptor endocytosis not only contributes to signaling, but is also the first step in the degradation of activated PARs, which irrevocably terminates their ability to signal. Thus, if cleaved PARs remain at the cell surface, how is signaling regulated?

### Phosphorylation

Most information about the regulation of PARs has been derived from studies of the canonical mechanisms of PAR activation. After cleavage by thrombin or trypsin, both PAR_1_ and PAR_2_ are rapidly phosphorylated by protein kinases, including GRKs, and second messenger kinases such as PKA and PKC (135–137). Phosphorylation within the C-terminal tail of the receptor serves as the primary mechanism to shut down G protein coupling and signaling. PAR_1_ is phosphorylated by GRK3 in *Xenopus laevis* oocytes and GRK5 in human endothelial cells (135, 137). Although the specific GRKs that phosphorylate PAR_2_ in native systems have not been identified, it is clear that PAR_2_ activation by trypsin recruits multiple GRKs in overexpression system (138). In terms of selectivity of certain GRKs over others, in endothelial cells GRK5 is the critical isoform that mediates thrombin-induced desensitization of PAR_1_ (137). GRK5 overexpression inhibits thrombin-induced Ca^2+^ signaling whereas GRK3 and GRK6 have no such effect (137). Whether proteases that cleave PARs at other sites also induce GRK recruitment and differential receptor phosphorylation remains to be determined. However, given the inability of many of these alternatively cleaved receptors to recruit arrestins, alterations in receptor phosphorylation are likely to occur. Studies of other GPCRs, such as β-adrenergic receptors (139) and opioid receptors (140), suggest that receptor phosphorylation occurs in an agonist-selective manner. For example, different biased agonists for β-adrenergic receptor trigger distinct patterns of receptor phosphorylation by different GRKs, which may establish a “barcode” that determines β-arrestin recruitment and functional responses (139). Whether this is also the case for PARs remains to be explored.

Phosphorylation of GPCRs often leads to β-arrestin recruitment and receptor internalization. However, this is not always the case. The morphine-activated μ-opioid receptor is phosphorylated by GRK5 at Ser^375^, which is sufficient for receptor desensitization but not β-arrestin recruitment or receptor internalization. On the other hand, DAMGO, another agonist for the same receptor, leads to phosphorylation at both Ser^375^ and Thr^370^, which leads to both receptor desensitization and internalization (141). Thus, biased proteases signaling might be terminated by phosphorylation without necessary internalization. It will be of interest to determine whether different proteases induce specific patterns of receptor phosphorylation, and to determine the functional relevance of these events.

### Intracellular trafficking and signaling

β-Arrestins not only act as chaperone proteins that direct receptor trafficking, but also are active participants of signaling by internalized receptors. β-arrestins mediate multiple steps of PAR signaling, including PAR_1_-mediated Akt activation, and PAR_1_ and PAR_2_-dependent ERK1/2 activation (96). After activation by trypsin, PAR_2_ stably couples to β-arrestin and together they co-translocate to early endosomes where they generate a second wave of intracellular signals (96). As an important scaffolding protein, β-arrestin is essential for the formation of the signaling complex including PAR_2_-Raf1 and activated ERK. This complex will ensure the appropriate subcellular localization of PAR_2_-mediated ERK activity. Thus, the stability between activated receptor and β-arrestin is essential for determining the duration of this activation. It has been pointed out that PAR_2_ may induce distinct cellular response from Gαq pathway via a β-arrestin-mediated mechanism (96, 142, 143). However, proteases such as elastase and cathepsin S are unable to induce β-arrestin recruitment, suggesting their lack of ability to further promote β-arrestin-dependent signals. In contrast to PAR_2_, where both β-arrestins have similar effects, PAR_1_-mediated Akt signaling is differentially mediated by different β-arrestins, depending on the mechanism of proteolytic activation. For example, β-arrestin 1 is required for rapid activation of Akt induced by thrombin, whereas APC cleavage leads to β-arrestin 2-dependent Akt activation (53, 134, 144). Although the underlying mechanism is not established, it may relate to different receptor conformations.

Besides desensitization, receptor trafficking to different subcellular compartments also plays an important part in regulation of GPCR signaling. For both PAR_1_ and PAR_2_, activation by trypsin or thrombin leads to receptor trafficking to endosomes, followed by lysosome sorting and receptor down-regulation. (145). However, many proteases such as elastase and APC failed to induce PARs endocytosis. The regulatory machinery for biased protease-signaling remains unknown. The potential involvement of compartmentalization and redistribution of the receptors to membrane subdomains seems to be an attractive area to explore. As mentioned earlier, APC-induced PAR_1_ signals require localization of the signalosome to caveolae, a specific lipid rich plasma membrane microdomain. Caveolae has also been suggested to be involved in TF-mediated PAR_2_ signaling. In breast carcinoma cells, both TF and PAR_2_ are observed co-localized in cholesterol-rich caveolae, and depletion or sequestration of plasma membrane cholesterol significantly impaired TF-VII1 induced cell signaling (146). It would be interesting to see if different proteases prefer targeting PARs at certain membrane microdomains or there is PARs redistribution upon activation by different proteases.

## Translational Relevance of Protease-Biased Signaling of PARs

The contribution of PARs to important patho-physiological processes, including hemostasis, inflammation, pain, and proliferation, has been extensively investigated through studies of PAR-deficient mice and by use of proteases and synthetic agonists/antagonists of the canonical signaling pathways (21, 22). The capacity of certain proteases to acts as biased PAR agonists or even antagonists adds further complexity to this system, and the relevance of protease-biased signals to complex patho-physiological processes is far from clear. A major difficulty relates to the identities of the proteases that activate PARs under physiological conditions and during disease. Since proteases are regulated through post-translational control of activity (e.g., by zymogen processing and endogenous inhibitors), studies should include assessment of enzymatic activity rather than gene or protein expression. A major advance in this regard is the use of activity-based probes that covalently interact with activated proteases, allowing their localization by whole animal or cellular imaging and identification by proteomic approaches (99). This approach has been used to detect activated cathepsin S in macrophages of tumors and the inflamed colon, as well as in spinal microglial cells during colitis (67, 147). However, the use of probes is likely to reveal that multiple proteases become activated during physiological and pathological events, many of which could activate or disarm PARs. Additional information can be provided by studies of protease knockout mice or through use of selective inhibitors. However, a detailed understanding of the importance of biased signaling of PARs would probably require genetic or pharmacological strategies to selectively disrupt particular biased pathways, and such tools are currently lacking.

Although the patho-physiological importance of biased signaling of PARs is not fully understood, biased agonism could explain certain paradoxes about the patho-physiological contribution of PARs. For example, PARs can have both pro-inflammatory and anti-inflammatory roles, which may depend on the animal models, species, tissues, or the protease that drive the response. In an ovalbumin-induced model of allergic inflammation of the mouse airway, PAR_2_ deletion is protective, suggesting that PAR_2_ contributes to the development of immunity and to allergic inflammation of the airway (148). However, in a lipopolysaccharide-induced pulmonary neutrophilia model, PAR_2_ shows a protective effect (149, 150). The underlying mechanism of these observed differences is unclear. However, differences in the repertoire of proteases that are activated in acute versus chronic inflammation, leading to distinct mechanisms of PAR signaling, could be one explanation. Lipopolysaccharide-induced pulmonary neutrophilia is an acute inflammation characterized by influx of neutrophils and activation of elastase and proteinases 3, biased agonists of PAR_1_ and PAR_2_. On the other hand, ovalbumin-induced inflammation is characterized by infiltration of eosinophil and macrophages, leading to activation of distinct proteases (151). Thus, the predominant active proteases for PAR_2_ may be different in these two models, which could potentially activate different signaling pathways that lead to opposite responses. The contrasting pro-inflammatory and cytoprotective actions of thrombin and APC, respectively, may also be attributed to PAR_1_ biased signaling. In this instance, the relative concentration of the proteases as well as the occupancy of EPCR by its ligand are critical in determining the PAR_1_ signaling pathways (28).

## Conclusion and Future Directions

Considerable progress has been made in defining the mechanisms by which proteases and synthetic agonists activate PARs. Proteases, peptides, and small molecules have been identified that can activate PARs by distinct mechanisms, leading to the stimulation of divergent pathways of receptor signaling and trafficking. The information derived from these studies has provided insights into the signaling pathways that are responsible for certain patho-physiological processes.

However, there are many unanswered questions about biased signaling of PARs. The ability of a PAR cleaved by different proteases or bound to various synthetic agonists to differentially signal probably arises from distinct receptor conformations. However, the structures of PARs in these different stabilized states remain to be determined. Although some proteases activate PARs by exposure of a tethered ligand, this is not always the case and the mechanism by which proteolysis *per se* can activate signaling is unknown. There is tantalizing evidence to suggest that biased signaling may underlie contrasting patho-physiological consequences of PAR activation, depending on the available proteases and the nature of PAR signaling. However, the proteases that are responsible for PAR activation in particular cell types in different conditions remain to be identified, and the signaling pathways that give rise to particular patho-physiological outcomes are not fully defined. Finally, very little is known about the mechanisms that regulate protease-biased signaling of PARs, particularly by those proteases that fail to promote the recruitment of β-arrestins and endocytosis of the activated receptors.

Whether protease-biased signaling of PARs can be exploited therapeutically remains an open question. The development of receptor antagonists or agonists that target disease-relevant PAR signaling pathways without affecting beneficial signaling events could provide a route for enhanced selectivity, with fewer on target side-effects. Future challenges will be to identify the primary pathways that mediate PAR-dependent physiological and patho-physiological events, and to develop receptor agonists and antagonists that selectively target these pathways. A deeper understanding of the mechanisms of initiation, regulation, and termination of protease-signaling will have profound implication in developing therapeutics for many critical conditions, including sepsis, thrombosis, inflammation, and pain processes.

## Conflict of Interest Statement

The authors declare that the research was conducted in the absence of any commercial or financial relationships that could be construed as a potential conflict of interest.

## Abbreviations

AP, activating peptide; APC, activated protein C; EPCR, endothelial protein C receptor; ERK, extracellular signal-regulated kinase; F, coagulation factor; GPCR, G protein-coupled receptor; GRK, G protein-coupled receptor kinase; MAPK, mitogen-activated protein kinase; MMP, matrix metalloproteinase; PAR, protease-activated receptor; TF, tissue factor.
