# Corrigendum: Biased Signaling of Protease-Activated Receptors

**DOI:** 10.3389/fendo.2014.00228

**Published:** 2015-01-06

**Authors:** Peishen Zhao, Matthew Metcalf, Nigel W. Bunnett

**Affiliations:** ^1^Monash Institute of Pharmaceutical Sciences, Parkville, VIC, Australia; ^2^Department of Pharmacology, University of Melbourne, Melbourne, VIC, Australia

**Keywords:** PARs, proteases, biased signaling, G proteins, β-arrestins, signal transduction

Figure 3 shows a snake diagram of the N-terminal amino acid sequence of human protease-activated receptor (PAR2). It indicates the sites at which different proteases cleave PAR2. These sites are correct. The error is that we accidentally repeated a sequence of amino acid residues (11–16, GAAILL) in positions 17–22 of the snake diagram. We wish to replace the snake diagram with the correct version.

**Figure 3:**
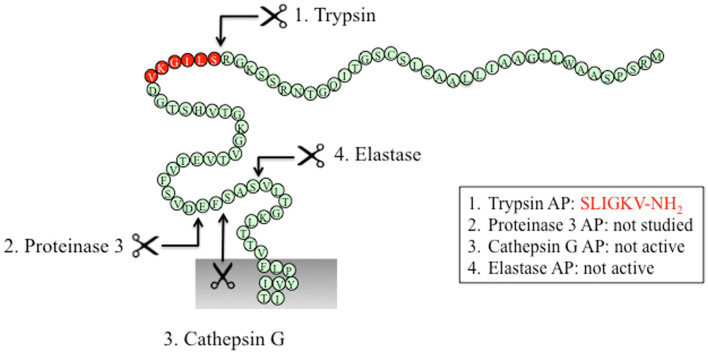
(corrected).

This minor correction does not affect our discussion or conclusions.

## Conflict of Interest Statement

The authors declare that the research was conducted in the absence of any commercial or financial relationships that could be construed as a potential conflict of interest.

